# The shaping of mRNA translation plasticity by RNA G-quadruplexes in cancer progression and therapy resistance

**DOI:** 10.1093/narcan/zcae025

**Published:** 2024-05-31

**Authors:** Anne Cammas, Alice Desprairies, Erik Dassi, Stefania Millevoi

**Affiliations:** Centre de Recherches en Cancérologie de Toulouse (CRCT), Equipe Labellisée Fondation ARC, Université de Toulouse, Inserm U1037, CNRS, 2 avenue Hubert Curien, 31037 Toulouse, France; Centre de Recherches en Cancérologie de Toulouse (CRCT), Equipe Labellisée Fondation ARC, Université de Toulouse, Inserm U1037, CNRS, 2 avenue Hubert Curien, 31037 Toulouse, France; Laboratory of RNA Regulatory Networks, Department of Cellular, Computational and Integrative Biology (CIBIO), University of Trento, Via Sommarive 9, 38123 Trento (TN), Italy; Centre de Recherches en Cancérologie de Toulouse (CRCT), Equipe Labellisée Fondation ARC, Université de Toulouse, Inserm U1037, CNRS, 2 avenue Hubert Curien, 31037 Toulouse, France

## Abstract

Translational reprogramming in response to oncogenic signaling or microenvironmental stress factors shapes the proteome of cancer cells, enabling adaptation and phenotypic changes underlying cell plasticity, tumor progression and response to cancer therapy. Among the mechanisms regulating translation are RNA G-quadruplexes (RG4s), non-canonical four-stranded structures whose conformational modulation by small molecule ligands and RNA-binding proteins affects the expression of cancer proteins. Here, we discuss the role of RG4s in the regulation of mRNA translation by focusing on paradigmatic examples showing their contribution to adaptive mechanisms of mRNA translation in cancer.

## Graphical Abstract

**Graphical Abstract:**
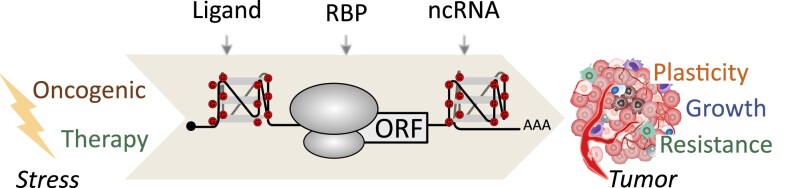

## Introduction

**Figure 1.**
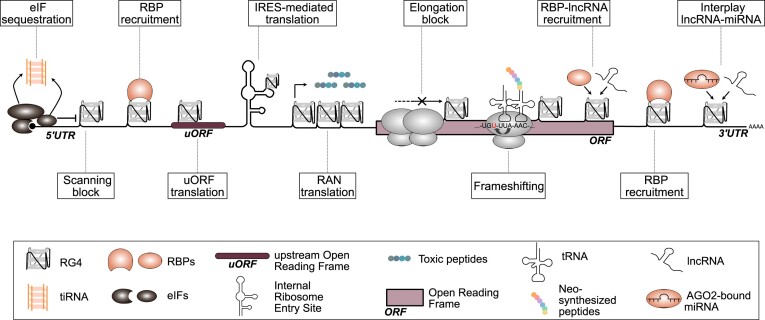
Molecular determinants driving RG4-dependent mRNA translation.

Recently, the concept of plasticity has been applied to mRNA translation regulation and dynamics in cancer cells (1). Indeed, this energy-consuming post-transcriptional step is capable of integrating oncogenic stimuli and signals from various endogenous and microenvironmental stressors to shape the proteome qualitatively, quantitatively, rapidly and dynamically. As a result of translational plasticity, the cell can adapt its needs to changing and challenging conditions, enabling it to survive by activating processes underlying cellular plasticity and promoting cancer onset, progression and resistance to anti-cancer therapies. Translational rewiring or reprogramming involves global regulation to reduce overall protein synthesis and thus energy consumption. Concurrently, it determines the selective activation or maintenance of the synthesis of proteins required to respond to cellular stimuli and stressors. Both require fine-tuned, adjustable regulation involving *trans*-acting factors, including RNA-binding proteins (RBPs) and non-coding RNAs. These factors act in concert with *cis-*acting elements, including sequence motifs, RNA structures and the translational machinery to decide if, when, where and how an mRNA should be translated. Among those *cis-*elements, RNA G-quadruplex structures (RG4s) are emerging as important players in the regulation of mRNA translation in cancer cells for several reasons (2). First, these structures are highly stable. They do not obey Watson–Crick's rules because guanines within G-tetrads are stabilized by Hoogsteen base pairing, and are therefore called non-canonical. At the same time, however, their folding is flexible depending on the cellular ions and protein factors (RBPs and helicases) or nucleic acids (DNA and RNA) with which they interact. A second key aspect is that, in addition to cellular factors, these structures can be targeted by small-molecule ligands capable of unfolding or stabilizing them. This has not only proved valuable for their identification and characterization in terms of structure–function relationships, but also holds promise for innovative anti-cancer strategies (3). The possibility of targeting RG4s for therapeutic purposes is compelling, since RG4s regulate the translation of factors associated with every cancer trait and modulate cancer cell pathways (2,4). RG4s are enriched in untranslated regions (UTRs) (5,6) and can also play a role when present in open reading frames (ORFs) (5,7). However, position matters when it comes to how RG4s control translation [(2,4) and references therein) (Figure 1). Indeed, RG4s located in the 5′UTR can repress cap-dependent protein synthesis by preventing the assembly of the translation initiation machinery on the mRNA. Alternatively, they can slow down scanning, thus inducing the accumulation of the small ribosome subunits (43S) in the 5′UTR. RG4s found in the ORF have instead been associated with ribosomal pausing and dissociation as a mechanism with a similar outcome or with ribosome frameshifting. Finally, RG4s in the 3′UTR can act as activators or repressors of cap-dependent translation via RBPs or microRNAs (miRNAs). The presence of other elements in the mRNA can also contribute to the impact of RG4s on translation. For example, although controversial, a translation-enhancing effect, via modulation of secondary structure, was observed instead of translation repression for RG4 5′UTRs located near an internal ribosome entry site (IRES). On the other hand, the presence of a short upstream ORF (uORF) next to a 5′UTR RG4 can promote the uORF translation, consequently repressing the translation of the downstream coding sequence (CDS). Moreover, RG4s formed by abnormal expansions of G-rich repeated sequence can increase translation initation at non-AUG codons. This phenomen, which is called repeat-associated non-AUG (RAN) translation, results in the production of toxic dipeptide repeat proteins The folding status of the RG4 is a fundamental determinant of the outcome of these mechanisms, implying a key role for RNA helicases and other factors that can modulate RG4 folding. In this review, we provide examples highlighting the importance of RG4 structures in mRNA translational plasticity and illustrate how these mechanisms shape cancer cell phenotypes linked to tumor development, dissemination and response to therapy.

## RG4s in translational plasticity

**Figure 2.**
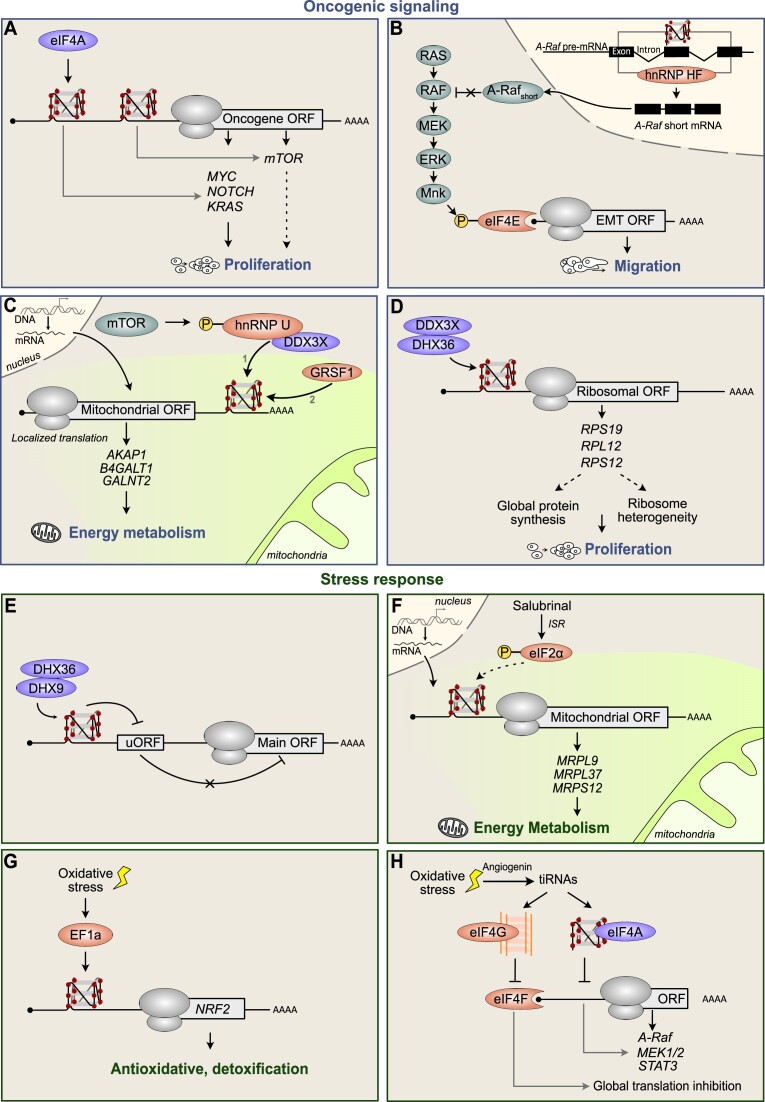
Upstream oncogenic and stress signals modulating RG4-mediated translation. Oncogenic signaling (A–D) and stress response pathways (E–H) modulate RG4 conformation and function in mRNA translation via the action of RG4-binding proteins (in orange) and helicases (in purple). (**A**) RG4s in oncogene 5′UTRs regulate mRNA translation with implication for proliferation. eIF4A may play a role in resolving RG4 structures. (**B**) RG4- and hnRNP H/F-dependent splicing regulation of *A-RAF* controls the RAF–MEK–ERK/MAPK pathway, resulting in eIF4E phosphorylation, which in turn modulates the translation of factors involved in EMT, with implication for cell migration. (**C**) mTOR controls the translation of some mitochondrial-localized nuclear-encoded mitochondrial mRNAs via hnRNP U phosphorylation. The underlying mechanism, similar to the ‘bind–unfold–lock’ model (16), involves DDX3X recruiting hnRNP U to folded RG4s (1), which in turn facilitates their unfolding and makes GRSF1 binding accessible (2). (**D**) RG4s in the 5′UTR of some ribosomal proteins (RP) inhibit mRNA translation, possibly inhibition of global protein synthesis and/or impacting on ribosome heterogeneity. DDX3X and DHX36 co-localize with RP RG4s and regulate their structure and function. (**E**) RG4s at uORFs increase DHX36/DHX9-dependent translation, which in turn down-regulates the translation of the main ORF (mORF). (**F**) Salubrinal-induced integrated stress response (ISR) via eIF2α increases the translation of nuclear-encoded mitochondrial mRNAs carrying predicted RG4s in their 5′UTR. (**G**) Oxidative stress induces the translation of the mRNA encoding NRF2, an anti-oxidant and detoxification factor, through increased binding eF1A to the 5′UTR RG4. (**H**) tRNA fragments generated by angiogenin-mediated cleavage under oxidative stress, called tiRNAs, can assemble to form inter-/intramolecular RG4s that bind either eIF4G to kick out the cap-binding complex or eIF4A, resulting in translation inhibition. In all the panels, the dotted and gray arrows represent links that remain to be fully demonstrated and describe mRNA-specific RG4-mediated translation mechanisms, respectively.

Aberrant oncogene signaling is one of the main drivers of plasticity in cancer cells. It promotes cell proliferation, inducing unfavorable cellular conditions due to a lack of nutrients and oxygen which the cell must overcome to survive. Activation of the PI3K/PTEN/AKT/mTOR and RAS/RAF/MEK/MNK/eIF4E signaling pathways alters the expression and activity of the eIF4F complex, formed by the cap-binding protein eIF4E, the RNA helicase eIF4A and the scaffolding protein eIF4G. In turn, the complex coordinates the selective translation of functionally related mRNAs playing a role in tumor development. RG4s are involved in these mechanisms at several levels. Indeed, RG4s may act upstream of oncogenic cascades by modulating the translation of oncogenes such as *MYC, MTOR*, *NOTCH*, *NRAS* and *KRAS* (8–10) (Figure 2A). The eIF4A helicase has been shown to be involved in the translation of some of these oncogenes in T-cell acute lymphoblastic leukemia, pancreatic adenocarcinoma and diffuse large B-cell lymphoma. It unwinds two-quartet RG4 structures formed by 12-nucleotide guanine (CGG)4 motifs, that were found enriched in the 5′UTRs of eIF4A-dependent mRNAs (10–12). Although the ability of these motifs to fold into two-quartet RG4s and underly eIF4A sensitivity to rocaglates was subsequently debated (13,14), three-layered RG4s in 5′UTRs cause translational repression and eIF4A dependency (14). The impact of RG4s on oncogenic signaling may also be indirect, modulating the expression of factors involved in signaling cascades through RG4-dependent regulation of other post-transcriptional steps. This is the case for the RG4 in the *A-RAF* pre-mRNA which, together with the heterogeneous nuclear ribonucleoprotein (hnRNP) H/F RBP, regulates the expression of an alternative spliced isoform of this transcript in glioblastoma (GBM) cells. This in turn modulates the RAF–MEK–ERK/MAPK signaling axis, changing eIF4E phosphorylation and subsequently affecting the synthesis of proteins involved in epithelial–mesenchymal transition (EMT) and metastasis, including SNAIL and MMP9 (15) (Figure 2B). Finally, oncogenic signaling pathways can directly modulate the conformation of RG4s by interacting with their effectors within the translation machinery. Indeed, recent findings show that mammalian target of rapamycin (mTOR) modifies the translation of some nuclear-encoded mitochondrial mRNAs by acting on the phosphorylation of the hnRNP U RBP, which binds RG4s in 3′UTRs. Similar to the previously proposed ‘bind–unfold–lock’ model [(16), see below], hnRNP U unfolds RG4s in synergy with the RG4 helicase DDX3X. The subsequent recruitment of GRSF1 then results in increased mRNA translation at the outer mitochondrial membrane and mitochondrial respiration (Figure 2C). This example provides the first link between oncogenic signaling, RG4-dependent translational regulation and the control of energy metabolism (17). RG4s may also modulate ribosome expression, which is required for the unrestricted growth of cancer cells and can be targeted by oncogenic signaling pathways. Indeed, RG4s have been identified in transcripts encoding ribosomal proteins (18). Their stabilization or unfolding by RG4 helicases (DDX3X and DHX36) or ligands modulates the translation of these transcripts, suggesting that RG4s can affect global protein synthesis (18) (Figure 2D). Many ribosomal proteins contain RG4s in their 5′UTRs (18), and a fraction of cytosolic ribosomes co-localize with RG4s (19). We can thus speculate that factors (ions, proteins and ligands) modulating RG4 conformation may play a role in ribosome heterogeneity (1) and in the formation of specialized ribosomes controlling the translation of specific mRNAs (20) (Figure 2D).

Beyond oncogenic signaling, stress generated by the microenvironment including genotoxic, oxidative, metabolic and proteotoxic stresses can trigger phenotypic plasticity through translational adjustments aimed at coping with stress and implementing a survival response. This involves the global inhibition of translation to preserve the cell's energy and the concomitant selective activation of mRNAs that function in stress response pathways (1,21). The molecular triggers may be common to both translation mechanisms, as in the case of the integrated stress response (ISR) (1). This leads to the phosphorylation of eIF2α to halt translation via inhibition of the formation of the eIF2–GTP–tRNA_i_Met ternary complex which joins the initiator tRNA^Met^ to the 43S pre-initiation complex. Also, it concurrently activates protein synthesis of specific mRNAs (1,21). This escape mechanism from a context of translation inhibition to express stress response proteins requires molecular determinants such as uORFs, whose inhibition of the translation of the downstream canonical main ORF (mORF) is relieved under stress conditions (1,21). RG4s were already shown to ‘rescue’ p53 tumor suppressor after genotoxic stress, when they recruit the cleavage/polyadenylation machinery via the hnRNP H/F RBP to maintain *p53* mRNA expression and function (22). In the case of mRNA translation, this role has not yet been fully demonstrated, but recent studies suggest that RG4s may contribute to stress-induced mRNA translational rewiring.

Indeed, RG4s located in uORFs were found to promote their translation and recruit the RG4 helicases DHX36 and DHX9, thus modulating the translation of mORFs encoding proto-oncogenes, transcription factors and epigenetic regulators (23) (Figure 2E). Whether RG4-containing uORFs contribute to selective protein synthesis in response to stress remains to be demonstrated. The involvement of RG4s in translational reprogramming has been proposed as a response to salubrinal, an ISR agonist, in melanoma. It was shown that, after stress exposure, two-thirds of the mRNAs still associated with ribosomes contained RG4s (24). Consistent with the observation that salubrinal dramatically increased mitochondrial translation, some nuclear-encoded mitochondrial mRNAs were found among the translated mRNAs, 80% of which contained RG4s (24) (Figure 2F). The ability of RG4s to rescue stress-sensitive mRNAs at the translational level has been demonstrated for NRF2, a transcription factor playing an antioxidant and detoxifying role and whose expression increases in response to oxidative stress. *NRF2* translation increases in response to H_2_O_2_ in an RG4-dependent manner. Although the proposed mechanism has not yet been fully demonstrated, oxidative stress does not alter RG4 conformation in the *NRF2* 5′UTR, in contrast to previous findings (25). It instead increases recruitment of the EF1a protein onto the *NRF2* RG4 (26) (Figure 2G).

RG4s also inhibit global translation by a mechanism that, independently of eIF2α phosphorylation, involves stress-induced cleavage of certain tRNAs to produce 5′ and 3′ tRNA-derived stress-induced RNAs (tiRNAs). Among them, 5′tiRNA^Ala^ and 5′tiRNA^Cys^ can assemble into unique tetramolecular RG4s (27) that associate with eIF4G, leading to cap-binding complex displacement and resulting in translational arrest (28). More recently, a similar mechanism involving tiRNA^Gln^ and eIF4A has been described to inhibit the translation of specific mRNAs, including *A-RAF*, *MEK1/2* and *STAT3*, and thus the progression of hepatocellular carcinoma (29) (Figure 2H).

Another mechanism by which RG4s may control mRNA translation in response to unfavorable ISR-activating conditions involves the formation of stress granules (SGs). SGs are clusters of ribonucleoproteins that form in response to different stresses and regulate multiple aspects of RNA metabolism, including mRNA stability and translation [(30) and references therein]. Indeed, 10% of cytoplasmic mRNAs with poor translation efficiency localize in SGs together with RBPs and translation factors. The assumption is that SGs contribute to translational repression by keeping non-translating mRNAs in a repressed state. However, recent studies propose that mRNAs in SGs can undergo translation (30). Evidence suggests a physical and functional link between RG4s and SGs [see (25) for a review]. Indeed, SGs assemble after the addition of RG4-forming exogenous RNA repeat sequences (31), or after RG4 stabilization following the down-regulation of RG4 helicases such as DHX36 (32), BLM (33) or DNAPTP6 (34). In addition, RG4s accumulate in SGs in response to stress and interact with the SG-nucleating protein G3BP1 (35). This is consistent with the observation that almost half of the mRNAs identified in SGs contain RG4s (35). Of note, DNA fragments generated after oxidative stress and forming G4s can also be found in SGs (36). Given these findings, and considering the involvement of RG4s in mRNA translation, it would not be surprising if RG4s were involved in regulating translation outside and inside SGs under conditions of SG induction [dependent or not (28) on eIF2α phosphorylation]. The observation that many types of stress, including oxidative stress, induce RG4 folding further suggests a role for these structures in the response to stress (25). The 3′UTRs would be mostly targeted by stress-induced RG4 formation, and some of them were shown to increase the stability of the transcripts in which they were contained. Yet, their effect on translation remains to be elucidated.

## RG4-mediated translation affects cancer cell plasticity

**Figure 3.**
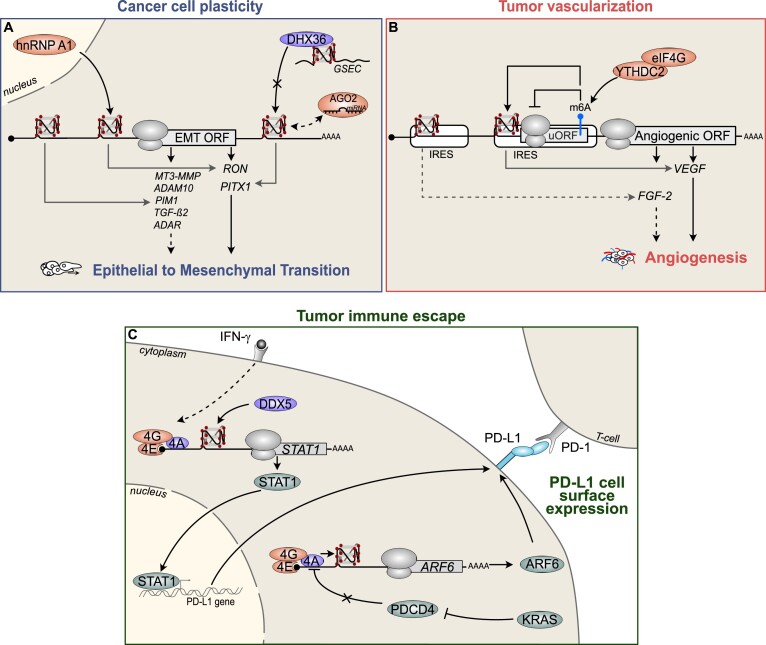
RG4s steer mRNA translation to shape cancer cell phenotypes. RG4-mediated translation impacts on cancer cell plasticity (A), tumoral microenvironment (TME) vascularization (B) and TME immunosurveillance (C). (**A**) 5′UTR- or 3′UTR-located RG4s regulate the translation of factors involved in the EMT. For RON, the RG4-mediated regulation of translation involves the cytoplasmic relocalization of the RBP hnRNP A1 and affects breast cancer cell migration. For PITX1, the binding of the RNA helicase DHX36 with the RG4 of the long non-coding RNA *GSEC* inhibits its activity as a translational regulator of PITX1 and impacts on colon cancer cell migration. Of note, PITX1 translation is regulated by an interplay between DHX36 bound to a 3′UTR RG4 and the miRNA machinery. (**B**) RG4s are located within IRESs of factors controlling angiogenesis. For VEGF, m^6^A methylation within the IRES favors the RG4-dependent translation while it inhibits the uORF-dependent translation through a mechanism involving the m^6^A reader YTHDC2 and eIF4G. This in turn promotes VEGF expression that is essential for angiogenesis in lung cancer. (**C**) RG4s modulate the translation of factors regulating PD-L1 cell surface expression. The 5′UTR RG4 of the transcription factor STAT1, known to control interferon-γ (IFN-γ)-induced PD-L1 expression, regulates its translation via the RNA helicases eIF4A (4A) and DDX5. ARF6, a small GTPase necessary for PD-L1 expression, contains an RG4 within the 5′UTR and is translationally regulated by eIF4A. The mutated KRAS inhibits the transcription of the eIF4A repressor PDCD4, thereby activating the RNA helicase activity of eIF4A and the translation of ARF6. 4A: eIF4A; 4E: eIF4E; 4G: eIF4G. In all the panels, the dotted and gray arrows represent links that remain to be fully demonstrated and describe mRNA-specific RG4-mediated translation mechanisms, respectively.

Cell plasticity enables cancer cells to adapt to their tumoral environment and escape the selective pressure of therapy through non-genetic mechanisms (1). This occurs through a reprogramming phenomenon that induces various phenotypic switches driving the dynamic acquisition of transient states. A notable example is the fluctuation between the epithelial and mesenchymal state, which confers advantages for tumor progression and resistance to treatments (1). Some evidence supports the view that RG4-mediated translation steers this phenotypic and functional plasticity by modulating the EMT. Indeed, 5′UTR-localized RG4s modulate the translation of mRNAs encoding factors involved in this cellular adaptative process, such as metalloproteinases (e.g. *MT3-MMP* and *ADAM10*), *PIM1*, *TGF-β2* and *ADAR* (Figure 3A). However, the consequences on EMT-related processes, such as cancer cell migration and invasion, remain to be fully investigated. This functional link has been demonstrated for RON, a tyrosine kinase known for its function in cellular motility and invasion. Indeed, the RG4 within the *RON* 5′UTR interacts with the hnRNP A1 RBP to increase its translation. The cytoplasmic relocalization of hnRNP A1 in breast cancer cells activates the RG4-dependent translation of *RON* and increases breast cancer cell migration. This demonstrates the impact of RG4-mediated translation in shaping the breast cancer cell migratory phenotype (Figure 3A) (37). Another example involves the antagonizing interplay between the RG4-containing *GSEC* long non-coding RNA and DHX36, that controls *PITX1* mRNA translation and colon cancer cell migration (38) (Figure 3A). Of note, DHX36-mediated binding to an RG4 located in the 3′UTR regulates the translation of the pro-migratory factor *PITX1*, together with the miRNA machinery (39) (Figure 3A). It remains to be established whether this functional link is mediated by PITX1 expression or by other DHX36 translational targets. Overall, these studies support the notion that RG4-dependent translational reprogramming of EMT-related factors leads to an adaptive cancer cell migratory phenotype.

## RG4-mediated translation regulates the tumoral microenvironment

Surrounding the tumor, blood vessel vascularization and immune cell infiltration evolve dynamically to create a tumoral microenvironment (TME) that is in constant crosstalk with cancer cells to influence their growth or response to treatments. Evidence linking RG4-mediated translation with angiogenesis or cell immune evasion controlled by the expression of the immune checkpoint shed light on a possible role for RG4s in TME remodeling. Indeed, although a direct functional impact on angiogenesis remains to be demonstrated, several studies suggest that RG4-mediated translation sustains vascularization by controlling the IRES-dependent translation of mRNAs encoding angiogenic factors such as *VEGF* (40,41) or *FGF-2* (42) (Figure 3B). Recently, it has been shown that the activating role of the *VEGF* RG4 in IRES-mediated translation is essential for angiogenesis in lung cancer (43). The underlying molecular mechanism involves m^6^A methylation within the *VEGF* IRES. This modification accelerates the proangiogenic effect of lung cancer, possibly through uORF inhibition, while facilitating RG4-mediated translation by recruiting the 48S pre-initiation complex mediated by the m^6^A reader YTHDC2 and eIF4G (Figure 3B). This study supports the emerging notion of an interplay between RG4s and epitranscriptomic modifications (44). The ‘switchable’ nature of the *VEGF* RG4, which can be modulated by RG4 ligands [i.e. Phen-DC(6) (40), oxymatrine (45)] or by RBPs [i.e. RBM4 (46)], paves the way for therapeutic strategies targeting *VEGF* mRNA expression to modulate TME angiogenesis.

RG4s may play a role in the TME by modulating the translation of factors regulating the expression of PD-L1. PD-L1 is a key component of tumor immunosuppression and a major target of immunotherapies aimed at reactivating the immune system to eliminate cancer cells. Indeed, in melanoma cells, the RG4 within the 5′UTR of *STAT1* mRNA inhibits the translation of this factor, known to induce PD-L1 transcription (47). This RG4-mediated mechanism is under the control of eIF4A. In turn, eIF4A can be targeted by silvestrol, eventually inhibiting the interferon (IFN)-γ-inducible cell surface expression of PD-L1 and the immune system (Figure 3C). The role of the *STAT1* RG4 in this regulatory axis remains to be characterized. However, the observation that eIF4A inhibition counteracts the induction of PD-L1 by IFN-γ in melanoma, breast and colon cancer cell lines suggests the *STAT1* RG4 to be a broad immunomodulator of PD-L1 expression in these cancer types. This role may also be considered for hepatocellular carcinomas in which the involved RNA helicase is instead DDX5 (48) (Figure 3C). Another example of RG4-mediated translation controlling PD-L1 expression and function was demonstrated in pancreatic ductal adenocarcinoma (PDAC) cells. (49) In this model, KRAS controls the RG4-mediated translation of *ARF6*, a small GTPase that is crucial for the immune evasion of PDAC cells. This occurs via the KRAS-mediated activation of eIF4A, obtained by transcriptional inhibition of the eIF4A repressor PDCD4 (Figure 3C) (49). Whether the *ARF6* RG4 impacts PDAC immune escape or ARF6-mediated mesenchymal programs driving PDAC cell plasticity (49) remains to be fully investigated. Taken together, these studies support the hypothesis that RG4-induced translational control of angiogenic factors or an inhibitory immune checkpoint is linked to angiogenesis and immune cell activity impacting TME vascularization and immunosurveillance.

## Targeting RG4-mediated mRNA translation to potentiate cancer therapy

The efficacy of cancer therapies is challenged by the emergence of therapeutic resistance mechanisms, leading to tumor relapse and poor patient outcome. Translation reprogramming stands out as a key driver of therapy resistance, providing a rationale for combining mRNA translation targeting with traditional cancer drugs to overcome resistance (1). Evidence showing that RG4s control the translation of factors involved in therapeutic resistance put those structures at the heart of the link between translation and resistance to cancer therapy. Targeting them in combination with standard drugs is therefore of great interest. Indeed, RG4s regulate the expression of factors involved in the DNA damage response (DDR), thereby impacting the response to genotoxic therapy. In GBM, several DDR factors were shown to be translationally regulated by 5′UTR RG4s. The underlying three-step ‘bind–unfold–lock’ regulatory mechanism involves: (i) hnRNP H/F being recruited by DHX36 to RG4s; (ii) RG4s unfolding; and (iii) RG4s being kept unfolded via hnRNP H/F binding, thereby increasing the expression of these DNA repair factors (16). Importantly, targeting this regulatory mechanism interferes with DDR protein expression, resulting in cellular DNA damage stress which sensitizes radio- and chemo-resistant GBM cancer cells to radiotherapy and temozolomide. Therefore, targeting RG4-dependent mechanisms to overcome radio-resistance in GBM cells has a considerable potential. This is also underscored by the cytotoxic effect of the ligand RHPS4 on glioblastoma stem-like cells (GSCs) known to be drivers of resistance in this cancer (50). The anti-proliferative effect of RHPS4 on GSCs is due to a decrease in the protein level of the DDR factor CHK1, without changes in the mRNA level, thus implicating translational regulation. Concomitantly, RAD51 transcription is inhibited, eventually resulting in a replicative stress and cell cycle blockage (50). Although the underlying molecular mechanism remains to be fully demonstrated, the *in silico* prediction of RG4s in CHK1 5′/3′UTRs (51) suggests their involvement in *CHK1*mRNA translation. Overall, these studies link RG4-mediated translation to genomic instability and therapy resistance in GBM.

Targeting RG4s to overcome resistance mechanisms has been demonstrated for the anti-cancer drug 5-fluorouracil (5-FU), an inhibitor of the thymidylate synthase (TYMS) enzyme involved in DNA synthesis. An RG4 in the 5′UTR of *TYMS* mRNA, interacting with TYMS itself, allows this factor to negatively autoregulate its protein synthesis (52). It has been proposed that TYMS inhibitors, such as 5-FU, prevent this interaction with its own RG4, thus increasing its expression and leading to resistance to inhibitors (52). Interestingly, targeting the *TYMS* RG4 with the polypurine reverse Hoogsteen hairpins oligonucleotide competes for the binding of TYMS to its own RG4 and increases the folding of this structure. This results in a decrease in TYMS protein levels that affects cell viability in synergy with 5-FU treatment in cervical carcinoma and prostate adenocarcinoma cells (52). Another example is the resistance to tumor necrosis factor-related apoptosis-inducing ligand (TRAIL)-induced apoptosis in breast cancer. The onset of this resistance is mainly due to the loss of the cell surface expression of its receptor (TRAIL-R2), mediated by its transcriptional repressor MAGED2 (53). The molecular mechanism controlling MAGED2 expression at the onset of TRAIL resistance involves a 5′UTR RG4 interacting with the RG4 helicase DDX21 (53), which unwinds this structure and activates *MAGED2* translation (53). Interestingly, inhibiting DDX21-mediated RG4-dependent translation of *MAGED2* correlates with an increase in TRAIL-R2 expression and a sensitization of breast cancer cells to TRAIL-mediated apoptosis. Taken together, these studies link RG4-dependent translation regulation and resistance to treatments in several types of cancers. This link underlines the potential therapeutic gains achievable by combining RG4 therapeutics with an existing cancer treatment.

## Conclusions

Evidence accumulated over the last 15 years strongly supports the notion that RG4s are key regulators of mRNA translation for functionally correlated mRNA subsets. This finding has important consequences for the cancer cell proteome and pathways impacting the cancer hallmarks. The examples presented here highlight that RG4-dependent translation is not only an effector of oncogenic signaling and stress response mechanisms. Indeed, it also contributes to the adaptive mechanisms that govern cancer cell plasticity and modulate tumor development and response to therapy. Future research will illustrate when, where and how RG4s link oncogenic signaling and endogenous/exogenous stress, including that induced by anti-cancer therapies, to cancer initiation, progression and resistance to therapy via mRNA translational regulation. As active research efforts focus on targeting RG4s and their clinical application (3), it would be important to determine whether strategies modulating RG4s are effective, independently or combined with treatments, in preventing and tackling resistance mechanisms.

## Data Availability

No new data were generated or analyzed in support of this study.
